# The use of pararectus approach for Type 3B Paprosky acetabular defect with intrapelvic cup migration. Case report

**DOI:** 10.1051/sicotj/2019008

**Published:** 2019-04-01

**Authors:** Mohammad K. Abdelnasser, Ahmed A. Khalifa, Yaser E. Khalifa, Hatem M. Bakr, Mohammad A. Mahran, Mohammad A. Moustafa, Ayman K. Mohammad, Ahmed M. Abdelaal

**Affiliations:** 1 Orthopedic Department, Assiut University Hospital Assiut Egypt; 2 General Surgery Department, Assiut University Hospital Assiut Egypt; 3 Orthopedic Department, Qena University Hospital Qena Egypt

**Keywords:** Pararectus approach, Intrapelvic cup, Acetabular defect

## Abstract

*Case*: A case of Type 3B Paprosky acetabular defect with intrapelvic cup migration where anterior column plating and cup extraction was done through an abdominal pararectus approach.

A male patient 63 years old reported progressive pain and walking disability after five years of cementless THR for right hip AVN.

CT pelvis showed loose intrapelvic migrated cup, extensive osteolytic acetabular defects, and pelvic discontinuity.

Pararectus approach was used to remove the cup and the head with concomitant plating of the anterior column

*Conclusion*: The pararectus approach is a valid option for intrapelvic cup extraction and pelvic discontinuity fixation.

## Introduction

Intrapelvic cup migration is a rare but serious complication after THR and many approaches have been described to deal with such complications [1,2].

The pararectus approach has been described as an excellent and less invasive alternative to other intraabdominal approaches for management of fractures of the acetabulum [3]. In this case report, we used the pararectus approach to manage a case with intrapelvic cup migration associated with pelvic discontinuity.

## Case report

An asthmatic male patient 63 years old presented to our University hospital with a severe groin pain and limitation of movement. Cementless THR on the right side was done for corticosteroid-induced AVN 7 years ago. Five years later, he experienced a progressive hip pain with a progressive decrease in walking ability.

**Figure 1.**
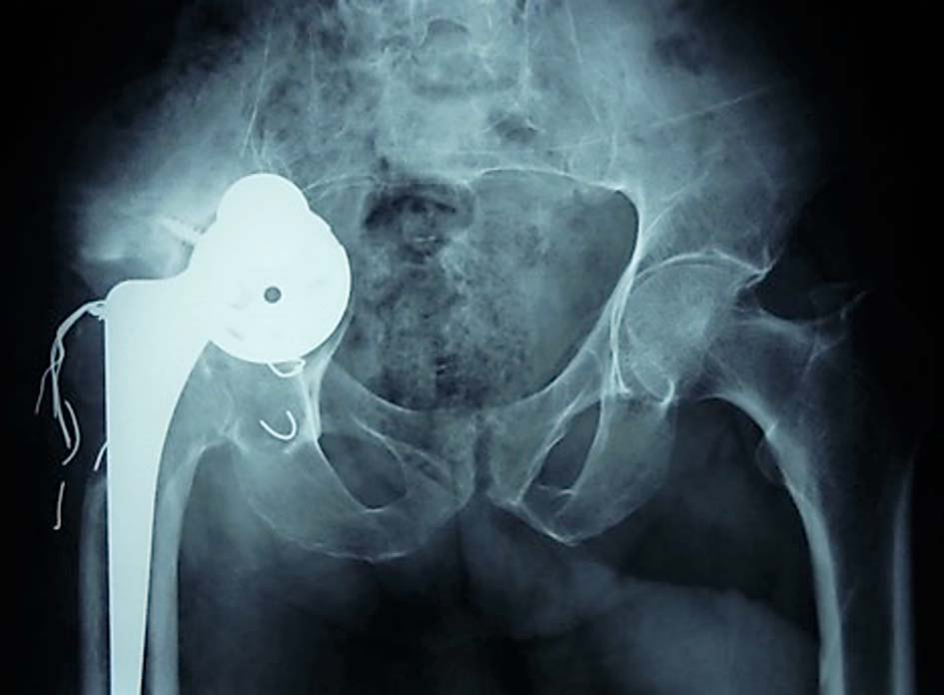
Anteroposterior X-ray of the pelvis showing acetabular component migration medial to Kohler’s line with proximal migration of the stem.

**Figure 2.**
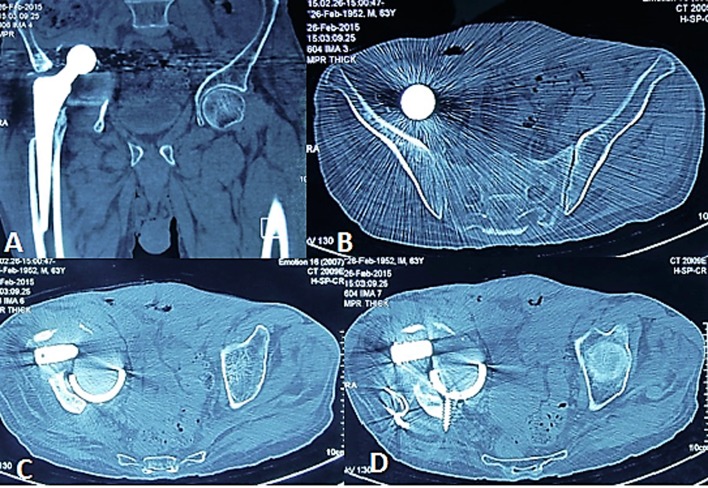
CT confirmed the intrapelvic protrusion of the cup, osteolytic defects, and the dome screw which is directed posteriorly.

AP X-ray of the pelvis showed loose cup with migration, medial to Kohler’s line and proximal migration of the stem (Figure 1). CT confirmed the intrapelvic protrusion and showed extensive osteolytic defect with suspicion of pelvic discontinuity (Figure 2).

**Figure 3.**
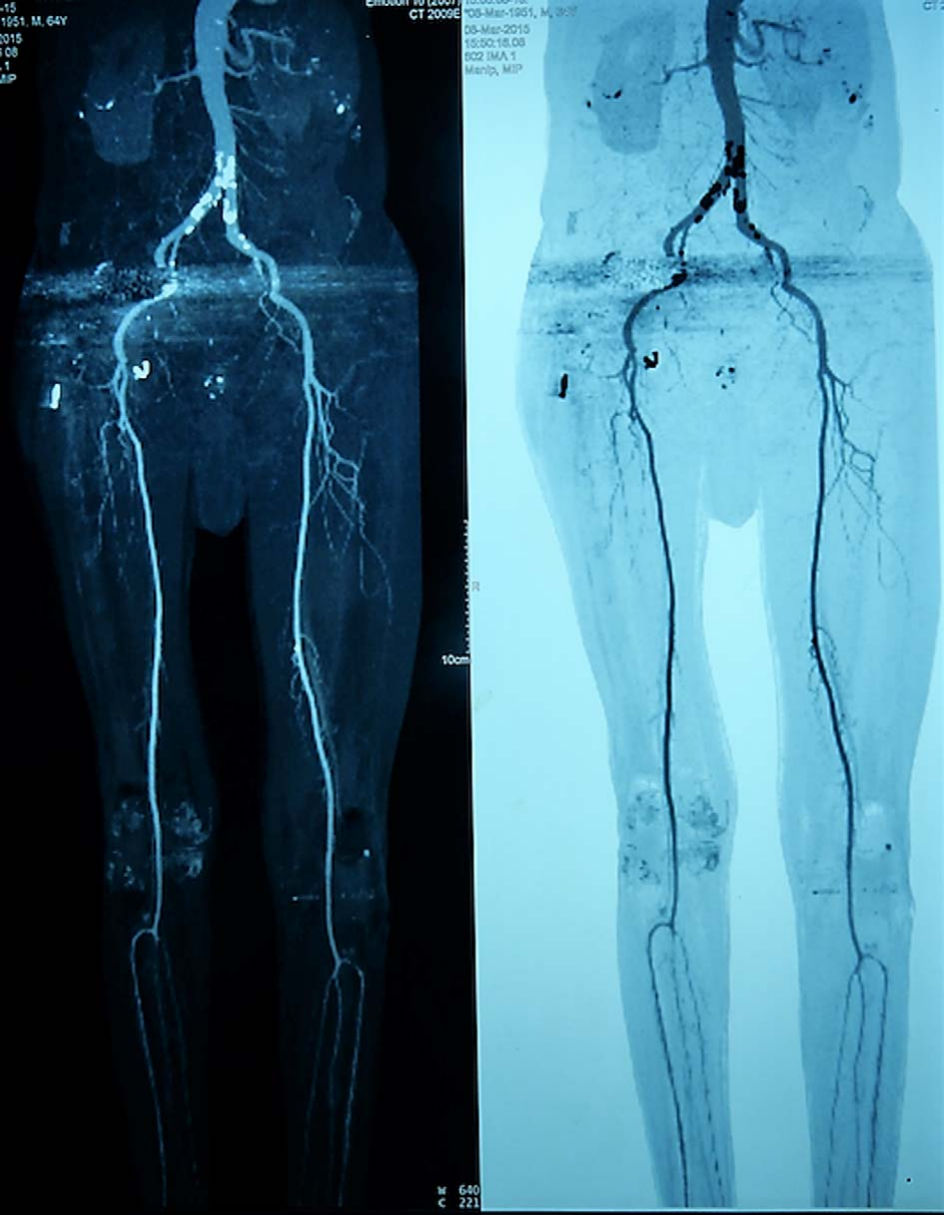
CT angiography showed mild displacement of the vessels with no evidence of arterial aneurysm.

CT angiography showed mild displacement of the external iliac vessels and excluded aneurysms (Figure 3).

The preoperative work-up (CBC, ESR, CRP), performed to exclude infection, was normal. We decided to go through a single-stage procedure, the pararectus approach for cup extraction and pelvis discontinuity fixation and a direct lateral approach for components’ revision.

With the assistance of a general surgeon, the pararectus approach was performed as described by Keel et al. [3]; the patient lied in the supine position and the skin incision is made starting from a point at the junction of the lateral and the middle thirds of the line connecting the umbilicus with the ASIS to a point at the junction of the middle and the medial thirds of a line connecting the ASIS with the symphysis. The subcutaneous fat and the deep layer of the fascia of the anterior abdominal wall are incised in line with skin incision. The rectus sheath is then incised at the lateral border of the rectus abdominus muscle. The fascia transversalis is incised longitudinally to enter the extraperitoneal space. The peritoneum is retracted craniomedially. Now, the external iliac artery and vein, the iliacus and psoas muscles with the femoral nerve, and the vas deferens are isolated and retracted using rubber catheters.

**Figure 4.**
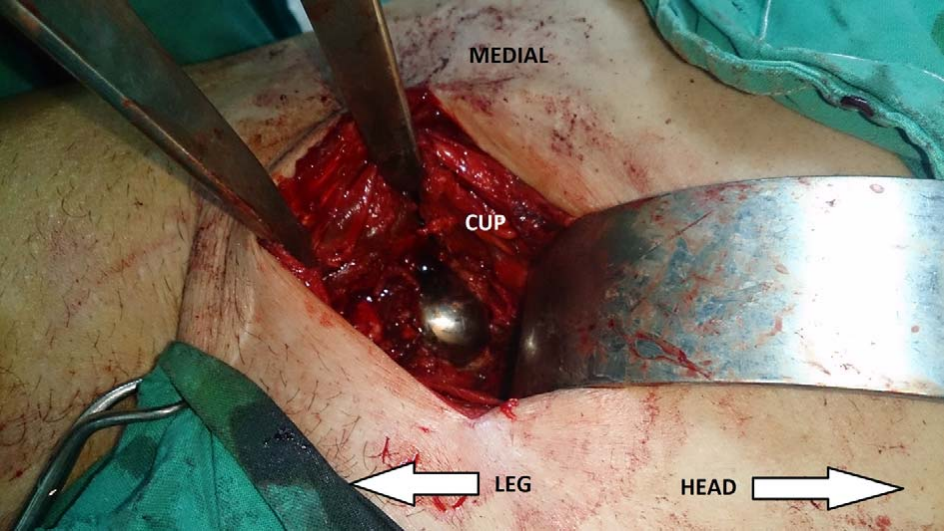
Intra-operative clinical photo of the pararectus approach showing the intra-pelvic cup before extraction.

**Figure 5.**
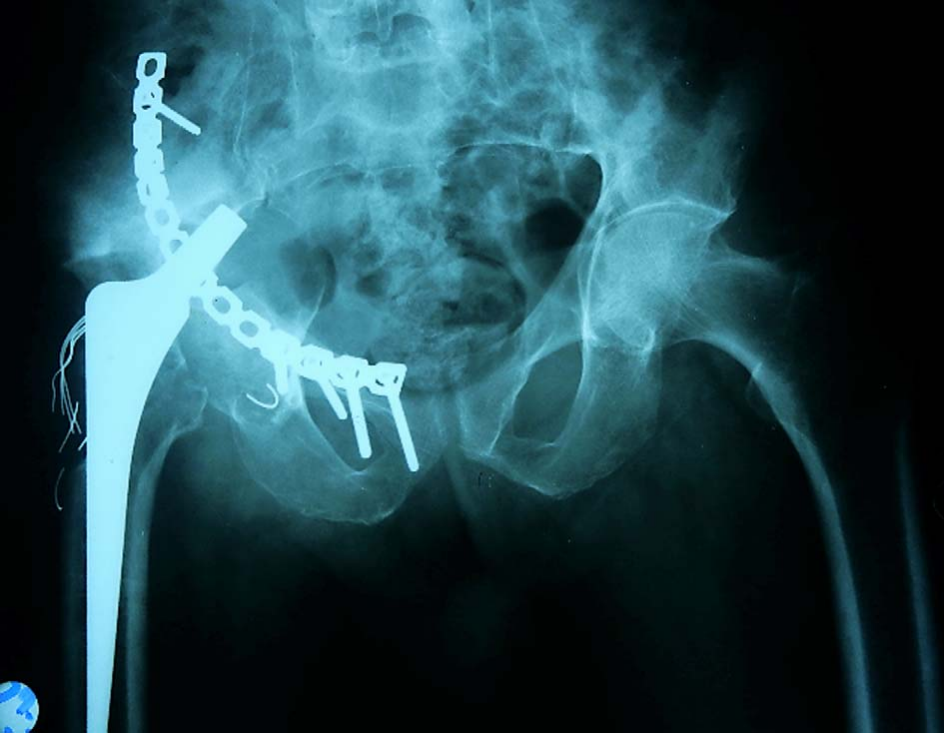
Intra-operative X-ray, the cup liner construct and the femoral head had been removed. Plating of the anterior column was done.

After isolation of the vessels and the vas, there was a thick fibrous membrane surrounding the cup and adherent to the vessels, as the usual in the case of chronic intrapelvic protrusion. After careful dissection from the vessels, the membrane was incised. The head was removed first to improve access to cup (Figure 4). Careful dissection was done around the dome screw to avoid injury of the ureter, then the cup was extracted safely. Then plating of the anterior column was done to fix the suspected pelvic discontinuity (Figure 5).

**Figure 6.**
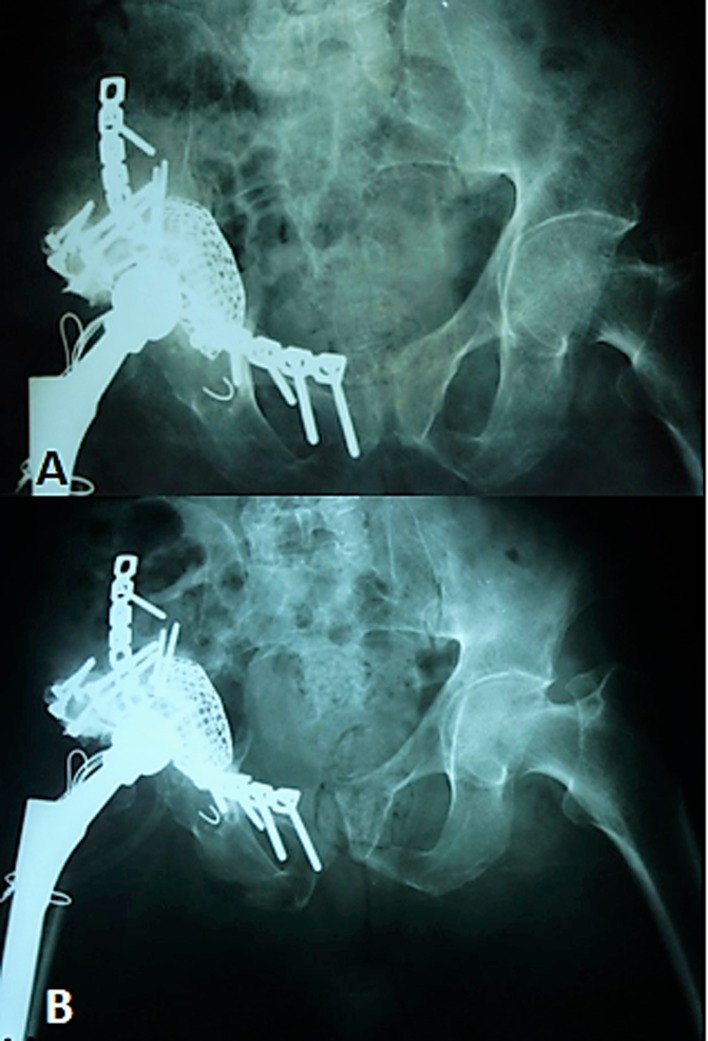
A-two year Postoperative follow up, B-immediate postoperative.

After closure of the pararectus approach, the patient was turned to the lateral decubitus position, and through a direct lateral approach, the stem was removed to access the acetabulum. Then the acetabulum was reconstructed with the use of floor and rim mesh with impaction grafting followed by stem revision (Figure 6). Three specimens for culture and sensitivity and a specimen for biopsy were taken from each side, which were proven to be negative later on. Toe touch only was allowed in the first three months postoperatively and then gradual weight bearing thereafter.

## Discussion

Loosening of the acetabular component with medial wall perforation and intrapelvic migration is rare, but can be potentially hazardous. False aneurysm of the femoral artery with potential life-threatening hemorrhage, nerve palsy, and compression of the pelvic viscera are among the possible complications if these structures are entrapped in the scar tissue [2].

Stiehl reported a high incidence of chronic infection accompanying intrapelvic cup migration. In his small cohort of seven cases, infection was the direct cause in three cases (43%) [4].

Many approaches were described to manage such problems. When protrusion is moderate, conventional approaches for THA can be used [5].

However, protrusion of the implant beyond the ilioinguinal line indicates displacement of the external iliac artery and femoral nerve. Therefore, Stiehl recommended using a double approach in these cases [4].

Grigoris et al. described a limited retroperitoneal approach to retrieve protruded acetabular cups [5]. However, this approach is not suitable for severe medial protrusion and does not safely isolate important pelvic structures. If bleeding occurs during component removal, a more formal approach is needed.

Stiehl used the anterior rectus retroperitoneal approach or the extensile triradiate approach with ilioinguinal extension to retrieve acetabular components [4]. He recommended keeping a high index of suspicion of infection in dealing with such cases. He also recommended the abdominal approach to be combined with the conventional approach to the hip to safely extract the protruded implant.

Chana-Rodríguez et al. used the Stoppa approach as an alternative for other extensive approaches to retrieve protruded acetabular cups [2]. One possible limitation to this approach is the possibility of a vascular injury when removing the cup screw construct, especially in the presence of dome screws necessitating dual approach to disengage the liner first, facilitating screw removal.

Keel et al. described the pararectus approach as an alternative to ilioinguinal and Stoppa approaches for the management of complex acetabular fractures [3]. It is less invasive than the ilioinguinal approach. In contrast to the ilioinguinal approach, there is no dissection of the inguinal canal. This protects against occurrence of inguinal hernias, which were reported with the ilioinguinal approach [3]. Secondly, neurovascular structures can be safely isolated and protected. Moreover, the pararectus approach allows direct access to the quadrilateral plate and anterior column. A possible limitation of this approach is the risk of peritoneal perforation. The pararectus approach may be difficult in obese patients or those with abdominal distension, ileus, or bowel obstruction [3].

In our case, we followed the preoperative protocols proposed by many authors including preoperative workup to exclude infection, (CBC, ESR, CRP), CT angiography to detect displacement of the vessels and to exclude the presence of aneurysm, and CT to accurately delineate the amount of pelvic osteolysis and to exclude the presence of pelvic discontinuity [1,4].

After extraction of the protruded acetabular component, plating for the anterior column was done to act as a bed support for the acetabular reconstruction and to stabilize pelvic discontinuity.

Acetabular reconstruction was done using a floor mesh for the defective medial wall and a roof mesh with impaction grafting; we preferred impaction grafting over other methods of reconstruction because of its biological potential and relatively low cost.

## Conclusion

Intrapelvic migration of the protruded acetabular component beyond Kohler’s line requires an intrapelvic approach for safe implant retrieval. The use of the pararectus approach gave us many advantages. It provided direct and central access to the quadrilateral plate and to the protruded component, the important structures were safely isolated, and enabled easy fixation of the anterior column in case of pelvic discontinuity. However, a second approach is needed to address the revision of acetabular and femoral components. A general surgeon trained in the intraabdominal approach should be a part of the surgical team especially in the first few cases of the learning curve.
